# Altered Innate Immune Responses in Neutrophils from Patients with Well- and Suboptimally Controlled Asthma

**DOI:** 10.1155/2015/219374

**Published:** 2015-11-18

**Authors:** Francesca S. M. Tang, Gloria J. Foxley, Peter G. Gibson, Janette K. Burgess, Katherine J. Baines, Brian G. Oliver

**Affiliations:** ^1^Woolcock Institute of Medical Research, The University of Sydney, Sydney, NSW 2006, Australia; ^2^Discipline of Pharmacology, School of Medical Sciences, Faculty of Medicine, The University of Sydney, Sydney, NSW 2006, Australia; ^3^Priority Research Centre for Asthma and Respiratory Disease, The University of Newcastle, Newcastle, NSW 2308, Australia; ^4^Department of Pathology and Medical Biology, University of Groningen, University Medical Centre Groningen, 9713GZ Groningen, Netherlands; ^5^School of Life Sciences, University of Technology Sydney, Sydney, NSW 2007, Australia

## Abstract

*Background*. Respiratory infections are a major cause of asthma exacerbations where neutrophilic inflammation dominates and is associated with steroid refractory asthma. Structural airway cells in asthma differ from nonasthmatics; however it is unknown if neutrophils differ. We investigated neutrophil immune responses in patients who have good (A_Good_) and suboptimal (A_Subopt_) asthma symptom control.* Methods*. Peripheral blood neutrophils from A_Good_ (ACQ < 0.75, *n* = 11), A_Subopt_ (ACQ > 0.75, *n* = 7), and healthy controls (HC) (*n* = 9) were stimulated with bacterial (LPS (1 *μ*g/mL), fMLF (100 nM)), and viral (imiquimod (3 *μ*g/mL), R848 (1.5 *μ*g/mL), and poly I:C (10 *μ*g/mL)) surrogates or live rhinovirus (RV) 16 (MOI1). Cell-free supernatant was collected after 1 h for neutrophil elastase (NE) and matrix metalloproteinase- (MMP-) 9 measurements or after 24 h for CXCL8 release.* Results*. Constitutive NE was enhanced in A_Good_ neutrophils compared to HC. fMLF stimulated neutrophils from A_Subopt_ but not A_Good_ produced 50% of HC levels. fMLF induced MMP-9 was impaired in A_Subopt_ and A_Good_ compared to HC. fMLF stimulated CXCL8 but not MMP-9 was positively correlated with FEV_1_ and FEV_1_/FVC. A_Subopt_ and A_Good_ responded similarly to other stimuli.* Conclusions*. Circulating neutrophils are different in asthma; however, this is likely to be related to airflow limitation rather than asthma control.

## 1. Introduction

Mainstay therapy for asthma is a combination of a long-acting *β*_2_ agonist to relax smooth muscle in the airways and a corticosteroid to reduce inflammation in the lungs [1]. However, even at high doses of these medications some patients remain unresponsive. These patients with uncontrolled or difficult to treat asthma often have a neutrophilic phenotype [2, 3]; however a group of patients with a steroid refractory eosinophilic phenotype also exist [4, 5]. These patients not only are unable to obtain symptom relief but also suffer from more frequent and severe exacerbations [6, 7].

Asthma exacerbations may be triggered by a number of provokers of which the most common are respiratory infections. Viral infections have been extensively studied and rhinovirus (RV) is the most commonly detected virus in exacerbating adults [8]. Bacterial infections are not as rigorously examined but appear to also be clinically significant [9]. Respiratory infections pose a high threat to patients suffering from uncontrolled asthma as they can trigger exacerbations that are often severe and leave the patient hospitalised with limited treatment options [10, 11].

Neutrophils are the most abundant immune cell in the body; their main effector role is to control infections. CXCL8 is a potent neutrophil chemoattractant, neutrophil elastase (NE) has potent antimicrobial properties, and matrix metalloproteinase- (MMP-) 9 is important in activating antimicrobial peptides, all of which are released by neutrophils. Transient neutrophilia and neutrophilic inflammation are a normal phase of the immune response to pathogens [12]; however, chronic airway inflammation occurs in stable asthma [2, 13, 14]. In a study of 205 patients, multivariate linear regression has shown no association of airway neutrophilia with corticosteroid use [15], and airway neutrophilia occurs in asthmatic patients who are corticosteroid naïve [16]. In addition, neutrophil numbers [2] and neutrophil inflammatory mediators such as CXCL8 [17], NE [18], and MMP-9 [19] are elevated in the airways of patients with severe asthma and these levels correlate with disease severity [2, 20].

It has been shown that structural cells, such as epithelial and smooth muscle cells, in the asthmatic airway are different compared to nonasthmatic cells in both morphology and function [21–24]. It is believed that these functional abnormalities drive other changes in the airways which give rise to the hallmarks of asthma. We previously found that circulating neutrophils from patients with asthma are altered in their response to the viral mimetic, R848 by producing elevated levels of CXCL8 [25], and expression quantitative trait loci mapping in neutrophils has found immune dysfunction trait associated variants [26]. However, to date it has not been investigated if neutrophil functions differ in patients with suboptimal symptom control despite taking moderate to high dose steroid therapy. Neutrophil dysfunction may occur in these patients which would, in part, account for the greater inflammatory mediator load in this group of patients.

Neutrophils are produced in the bone marrow and have a relatively short life span [27, 28]. Given our previous finding of different responses of lung versus circulating neutrophils [29], to ascertain if neutrophils are already different prior to entering the lung in well- and suboptimally controlled asthma we compared the response to both bacterial and viral mimetics in circulating neutrophils to avoid any potential confounding effects of the lung inflammatory environment. We hypothesised that neutrophils from patients with suboptimally controlled asthma have a defective innate immunity which may predispose to pathogen-induced exacerbations.

## 2. Materials and Methods

### 2.1. Volunteer Recruitment

The project was approved by the Human Research Ethics Committee, The University of Sydney, prior to commencement. Volunteers with doctor diagnosed asthma, stable disease, and no reported symptoms of respiratory infection were recruited into the study. Healthy control volunteers were also recruited. Participants were required to be over 18 years of age and be fluent in English. Exclusion criteria included if they were pregnant, were known to faint during venipuncture procedures, or had a blood borne infection or condition. All volunteers provided written informed consent and were asked to complete a standardised questionnaire regarding age, gender, asthma symptoms, asthma medication use, and smoking history. Patients also completed baseline spirometry for forced expiratory volume in 1 second (FEV_1_) and forced vital capacity (FVC). Participants with asthma were asked to withhold their short-acting *β*_2_ agonists for a minimum of 6 hours and 24 hours for long-acting *β*_2_ agonists and inhaled corticosteroids (ICS).

### 2.2. Categorisation of Asthmatics

Participants with asthma were stratified based on their asthma control questionnaire (ACQ) score [30]. A cut point of ACQ < 0.75 was used to identify well-controlled asthma [30]. Participants with suboptimal asthma control (ACQ ≥ 0.75) also had evidence of variable airflow limitation (PD_15_ < 15 mL hypertonic saline or standard challenge agent, change in postbronchodilator FEV_1_ > 12% of 200 mL, >12% peak flow variability over at least 1 week, or FEV_1_ variability >12% of two measurements within two months of each other) and were taking a minimum of GINA step 3 maintenance combination therapy. Patient information is provided in Tables 1 and 2.

### 2.3. Neutrophil Isolation

Neutrophils were isolated from peripheral blood collected from volunteers with and without asthma by a modified standard protocol [29, 31, 32]. Briefly, 40 mL of blood was mixed with 10 mL acid citrate dextrose (ACD), 10 mL of phosphate buffered saline (PBS) (Gibco, Carlsbad, CA, USA), and 6 mL of 10% dextran (MP Biomedicals, Santa Ana, USA) and left for 20 minutes for sedimentation to occur at room temperature. The top layer was removed, overlaid on Ficoll Paque-PLUS (GE Healthcare, Little Chalfont, UK), and centrifuged at 490 g for 10 minutes. The supernatant was discarded and the cell pellet of granulocytes was resuspended in sterile water for 30 seconds to lyse remaining red blood cells before osmolarity was reestablished with equal parts of 2x PBS. Cells were then incubated for 30 minutes at 4°C with CD16 magnetic beads (Miltenyi Biotec, Bergisch, Germany) before running through a magnetic column as per the manufacturer's instructions. Previous optimisation of the protocol showed typical purity was 99% or greater by a haematoxylin and eosin stain. The main contaminating cell was eosinophils (<1%).

### 2.4. RV16

RV16 was generously donated by Professor Sebastian Johnston, Imperial College, London, UK. RV16 was grown in HeLa cells by standard procedures and infectivity titre determined by a titration assay as described [33].

### 2.5. Stimulation of Neutrophils with Toll-Like Receptor (TLR) Agonists and RV16

Neutrophils were resuspended in 1% fetal bovine serum (FBS) (Glendarach Biologicals, Melbourne, Australia), 1% 1 M HEPES (Gibco), and 1% penicillin/streptomycin RPMI 1640 (Gibco) at 1 × 10^6^ cells/mL. Cells were left unstimulated (negative control) or stimulated with EC_50_ concentrations of each TLR agonist based on dose-response curves generated for CXCL8 release (data not shown): 1 *μ*g/mL LPS (Sigma Aldrich, St. Louis, MO, USA), 3 *μ*g/mL imiquimod (Invivogen, San Diego, USA), 1.5 *μ*g/mL R848 (Invivogen), and 10 *μ*g/mL poly I:C (Sigma Aldrich), except fMLF (Sigma Aldrich) (100 nM) which was based on previous reports [34]. Neutrophils were also stimulated with RV16 at a multiplicity of infection (MOI) of 1 infectious particle per cell as previously published [25, 35]. Cells were incubated at 37°C with 5% CO_2_ for 1 hour for NE and MMP-9 measurements or 24 hours for CXCL8 measurements. Cell-free supernatant and neutrophils cell pellets were collected and stored at −80°C for analysis.

### 2.6. CXCL8 Enzyme-Linked Immunosorbent Assay (ELISA)

CXCL8 production was measured using a sandwich ELISA in duplicate. Specific ELISA kits from R&D Systems (Minneapolis, USA) were used according to the manufacturer's instructions. Detection limit was 15.6 pg/mL.

### 2.7. Neutrophil Elastase (NE) Activity Assay

NE activity was measured in duplicate using a fluorescence assay from Cayman Chemicals (Ann-Arbor, USA) according to the manufacturer's instructions. Fluorescence readings from samples were compared to a standard curve of known concentrations of NE to determine the concentration. Detection limit was 3.1 ng/mL.

### 2.8. MMP-9 Zymography

A bicinchoninic acid assay (Sigma Aldrich) was run for all samples according to the manufacturer's instructions to obtain the total protein concentration. Zymography was carried out according to previously published methods [36]. Briefly, 200 ng of total protein was loaded into each lane of a 1% gelatin polyacrylamide gel. The gel was run and then proteinases were activated in a CaCl_2_ activation buffer overnight before staining with Coomassie brilliant blue dye. Bands were determined to be pro-MMP-9 using size markers and MMP-9 standards. Densitometry was performed with Carestream Molecular Imaging Software on images taken on a Kodak Image Station from Integrated Sciences (Chatswood, Australia) to determine the relative fold change compared to media control.

### 2.9. Data and Statistical Analysis

For statistical analysis, data was normalised (log_10_) before normality tests were conducted (Kolmogorov-Smirnov, D'Agostino and Pearson, and Shapiro-Wilk normality tests; GraphPad Prism 6). They were deemed to have a normal distribution if they passed one of the three normality tests. A paired *t*-test or one-way analysis of variance (ANOVA) with Dunnett's posttest was performed if the data followed a normal distribution or a Wilcoxon matched *t*-test or Friedman test with Dunn's multiple comparison test if data were nonparametric. Two-way ANOVA with Tukey's posttest was performed for comparisons between healthy controls, well-controlled asthmatic, and suboptimally controlled asthmatic. For some data sets, correlation analysis was performed. Significant changes were identified where *p* < 0.05.

## 3. Results

### 3.1. Patient Characteristics

The clinical characteristics for the study population are detailed in Tables 1 and 2. Patients were all age and gender matched with the mean age of approximately 60 years and each group consisted predominately of males. By definition, mean ACQ was different between the two asthma groups (good symptom control: mean 0.32, suboptimal symptom control: mean 1.33) There were no differences in FEV_1_% predicted between the three patient groups; however FEV_1_/FVC% ratio was significantly less in patients with well-controlled and suboptimally controlled asthma compared to healthy controls (Table 1). The majority of participants with well-controlled asthma were taking a short-acting *β*_2_ agonist (82%) (Table 1) with only 55% taking an ICS containing inhaler daily (Table 2). However, all but 1 patient in this group took combination therapy intermittently in the past 12 months (Table 2). All participants with suboptimal asthma symptom control were taking a short-acting *β*_2_ agonist (Table 1) along with combination therapy (Table 2).

### 3.2. Differential CXCL8 Release from Neutrophils from Asthmatics

**Figure 1:**
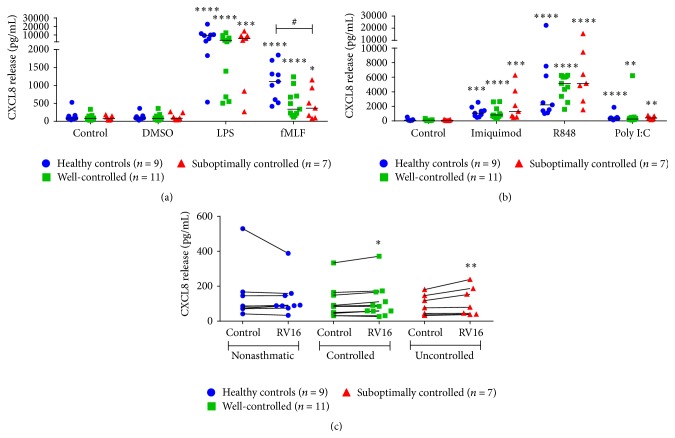
CXCL8 release from neutrophils stimulated with bacterial and viral mimetics and RV16. CXCL8 release from healthy controls (blue circles, *n* = 9), well-controlled asthmatic (green squares, *n* = 11), and suboptimally controlled asthmatic (red triangles, *n* = 7) neutrophils stimulated with (a) bacterial compounds: lipopolysaccharide (LPS), f-Met-Leu-Phe (fMLF), and DMSO (vehicle control), (b) viral surrogates: imiquimod, R848, and polyinosinic:polycytidylic acid (poly I:C), and (c) RV16 after 24 hours. Raw data is presented as a scatter plot with median. ^*∗*^ *p* < 0.05, ^*∗∗*^ *p* < 0.01, ^*∗∗∗*^ *p* < 0.001, and ^*∗∗∗∗*^ *p* < 0.0001 compared to unstimulated control. ^#^ *p* < 0.05 between indicated groups.

All bacterial and viral mimetics including LPS, fMLF, imiquimod, R848, and poly I:C induced significant CXCL8 release from neutrophils isolated from healthy controls and well-controlled and suboptimally controlled asthmatics (Figures 1(a) and 1(b)). Interestingly, RV16 induced CXCL8 only in neutrophils from well-controlled and suboptimally controlled asthmatics (Figure 1(c)). Neutrophils from suboptimally controlled asthmatics had a deficient CXCL8 response to fMLF of approximately half when compared to healthy controls (Figure 1(a)). There was also a trend for neutrophils derived from well-controlled asthmatics to release less CXCL8 in response to fMLF which was of similar magnitude to neutrophils from suboptimally controlled asthmatics (Figure 1(a)). All other stimulants induced similar production of CXCL8 between all three groups.

### 3.3. Differential NE Release from Neutrophils from Asthmatics

**Figure 2:**
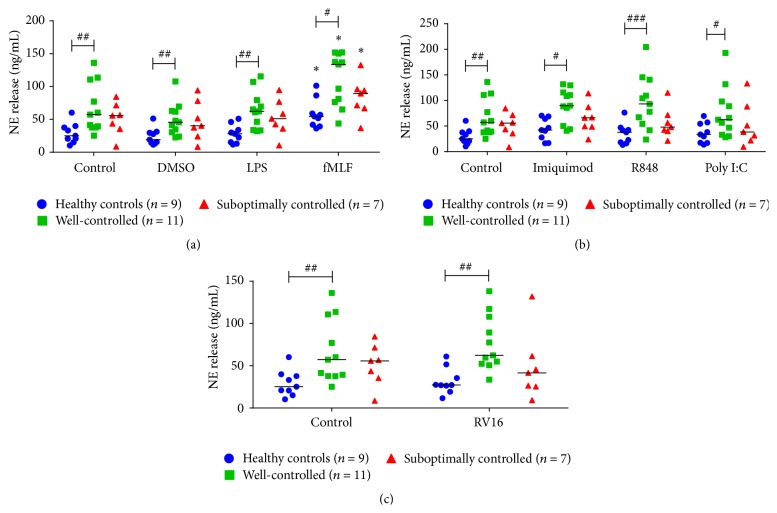
NE release from neutrophils stimulated with bacterial and viral mimetics and RV16. NE release from healthy controls (blue circles, *n* = 9), well-controlled asthmatic (green squares, *n* = 11), and suboptimally controlled asthmatic (red triangles, *n* = 7) neutrophils stimulated with (a) bacterial compounds: lipopolysaccharide (LPS), f-Met-Leu-Phe (fMLF), and DMSO (vehicle control), (b) viral surrogates: imiquimod, R848, and polyinosinic:polycytidylic acid (poly I:C), and (c) RV16 after 1 hour. Raw data is presented as a scatter plot with median. ^*∗*^ *p* < 0.05 compared to unstimulated control. ^#^ *p* < 0.05, ^##^ *p* < 0.01, and ^###^ *p* < 0.001 between indicated disease groups.

fMLF was the only pathogen mimetic to induce NE from neutrophils in all three groups (Figure 2(a)). Interestingly, we found that, at baseline, neutrophils from well-controlled asthmatics had enhanced NE release compared to healthy controls (Figure 2(a)). Furthermore, this difference was also found when stimulated with fMLF (Figure 2(a)). RV16 did not induce NE release from neutrophils in any of the three groups.

### 3.4. Differential MMP-9 Release from Neutrophils from Asthmatics

**Figure 3:**
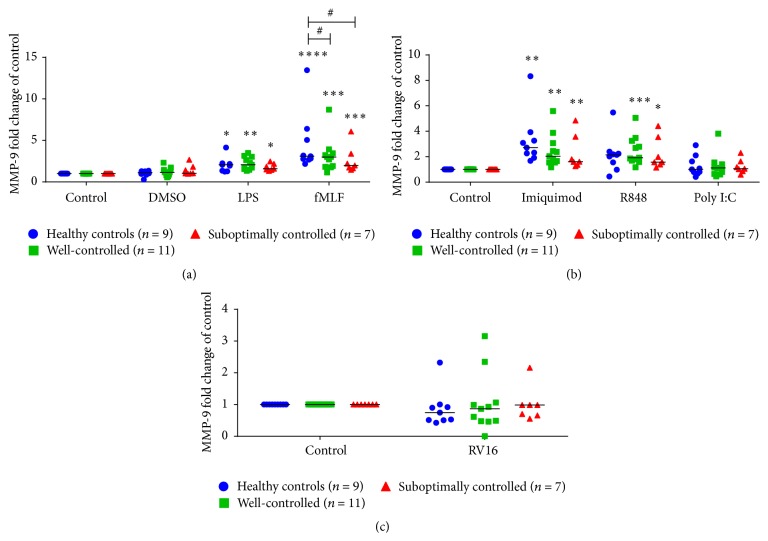
MMP-9 release from neutrophils stimulated with bacterial and viral mimetics and RV16. MMP-9 release from healthy controls (blue circles, *n* = 9), well-controlled asthmatic (green squares, *n* = 11), and suboptimally controlled asthmatic (red triangles, *n* = 7) neutrophils stimulated with (a) bacterial compounds: lipopolysaccharide (LPS), f-Met-Leu-Phe (fMLF), and DMSO (vehicle control), (b) viral surrogates: imiquimod, R848, and polyinosinic:polycytidylic acid (poly I:C), and (c) RV16 after 1 hour. Raw data is presented as a scatter plot with median. ^*∗*^ *p* < 0.05, ^*∗∗*^ *p* < 0.01, ^*∗∗∗*^ *p* < 0.001, and ^*∗∗∗∗*^ *p* < 0.0001 compared to unstimulated control. ^#^ *p* < 0.05 between indicated disease groups.

LPS, fMLF, and imiquimod induced MMP-9 release from neutrophils in all three groups (Figures 3(a) and 3(b)). Similar to CXCL8 data, fMLF stimulated neutrophils from well-controlled and suboptimally controlled asthmatics had a deficient MMP-9 response compared to healthy controls (Figure 3(a)). Interestingly, R848 selectively induced MMP-9 in neutrophils derived from well-controlled and suboptimally controlled asthmatics, but not in healthy controls (Figure 3(b)). RV16 did not induce MMP-9 release from neutrophils in any of the three groups investigated.

### 3.5. FEV_1_ % Predicted and FEV_1_/FVC% Ratio Correlated with fMLF Induced CXCL8

**Figure 4:**
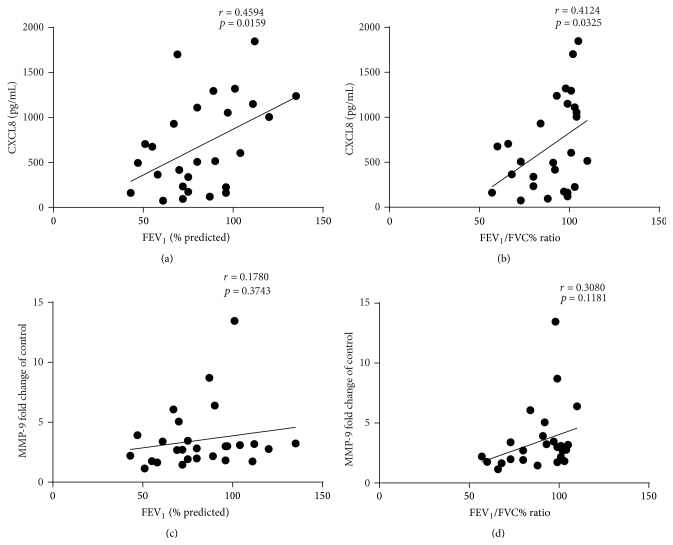
Correlation of FEV_1_ and FEV_1_/FVC with fMLF induced CXCL8 and MMP-9 from neutrophils. Correlations of fMLF induced (a-b) CXCL8 release and (c-d) MMP-9 with FEV_1_ (a-c) and FEV_1_/FVC ratio (b-d).

To further investigate if the differences in fMLF induced CXCL8 and MMP-9 in controlled and uncontrolled asthmatics were related to airway obstruction, we performed correlation analysis. We found that FEV_1_% predicted and FEV_1_/FVC% ratio positively correlated with fMLF induced CXCL8 release (Figures 4(a) and 4(b)) but not fMLF induced MMP-9 release (Figures 4(c) and 4(d)). There was no correlation between ACQ and fMLF induced CXCL8 (*r* = −0.318, 0.106) or MMP-9 (*r* = −0.181, *p* = 0.367). No correlations were found between pack year history and fMLF induced CXCL8 (*r* = −0.04, *p* = 0.84) or basal CXCL8 (*r* = −0.07, *p* = 0.74). Similarly, no correlations were found between daily ICS dose and fMLF induced CXCL8 (*r* = −0.15, *p* = 0.45) or basal CXCL8 (*r* = 0.12, *p* = 0.55). We also found no correlation between basal CXCL8 release and FEV_1_% predicted (*r* = 0.028, *p* = 0.889) or FEV_1_/FVC% ratio (*r* = 0.029, *p* = 0.884).

## 4. Discussion

In this study we found that neutrophils from patients with asthma respond differently to fMLF compared to healthy controls. However, we did not see differences between neutrophils from patients with well- versus suboptimally controlled asthma. RV16 induced CXCL8 and R848 induced MMP-9 occurred in only neutrophils from well- and suboptimally controlled asthmatic groups but not in neutrophils from healthy controls. fMLF stimulation resulted in a deficient MMP-9 production in neutrophils from well-controlled asthmatics and a deficient CXCL8 and MMP-9 in suboptimally controlled asthmatics. In addition, NE was differentially regulated and was constitutively elevated in well-controlled asthmatics. This increased constitutive release most likely accounts for the difference observed in fMLF stimulated neutrophils from well-controlled asthmatics. We also found that fMLF induced CXCL8 and MMP-9 release correlated with lung function but not ACQ, smoking history, or ICS use.

As airway epithelial cells and smooth muscle cells are fundamentally altered in asthma [21–24], even when grown for several cycles* in vitro *where they are deprived of altered signals in an asthmatic airway, we hypothesised that antimicrobial functions would similarly be dysfunctional in neutrophils from suboptimally controlled asthma. We hypothesised that there would be deficient immune responses in circulating neutrophils from people with suboptimally controlled asthma which potentially could lead to more severe or long lasting respiratory infections and ultimately an exacerbation. This study provides valuable and novel insights into circulating neutrophilic inflammatory responses.

In this study we were interested in investigating if neutrophils are already dysfunctional prior to entering the airway tissue in patients with asthma which could be intensified with altered inflammatory signals. As such, we chose to use peripheral blood neutrophils which we believe to be appropriate for investigation of possible differences in these cells. Since neutrophils are released into the circulation from the bone marrow we believe this cell population best reflects neutrophil function that has not had further differentiation signals provided during extravasation and in the airway lumen.

RV is a major precipitant of viral-induced exacerbations in asthma [8]. Neutrophils migrate into the airways during RV infections [38, 39] but their role in antiviral immunity remains unclear as is their ability to become infected with RV despite expressing ICAM-1 [40], the attachment protein for the serotype of RV used in this study. It is not clear why RV induced CXCL8 in this study. In the absence of replication we have previously shown that RV binding to ICAM-1 is sufficient to induce cytokine release in some [41] but not all lung cells [42]. Toll-like receptors (TLRs), particularly TLR 3, TLR 7, and TLR 8, detect viruses and usually their activation leads to typical innate activation. Potentially if RV is phagocytosed, TLR 3, TLR 7, and TLR 8 present on phagocytic vesicles may be activated. Alternatively cell surface TLR 3 [43, 44] may detect the presence of the virus.

Our finding that RV16 can induce CXCL8 in asthmatic neutrophils is novel, although the clinical relevance for the small induction observed here is questionable and needs to be interpreted with caution. However, the titre of RV* in vivo* is likely to be much higher than we used* in vitro* (reported 1000 TCID/mL in nasal lavage fluid [45]); therefore we speculate that a greater response to RV may occur* in vivo*. It is also plausible that patients with more severe disease are more sensitive to relatively small changes in inflammatory cytokine production due to the cumulative effect of a greater number of neutrophils present. Dysregulation in CXCL8 induction by RV16 could play a role in the pathogenesis of asthma exacerbations and persistent airway neutrophilia but this requires further investigation.

fMLF, a bacterial derived protein and ligand for the fMLF receptor, stimulates neutrophils to migrate, produce inflammatory mediators, and release granules and reactive oxygen species [46]. We observed stimulant and disease specific changes in the response to fMLF. We believe these changes are not related to fMLF receptor expression since in the same patients with well-controlled asthma; fMLF induced CXCL8 release was approximately half the response of healthy controls but NE release was augmented.

NE and MMP-9 are both proteases and are found in neutrophil azurophil and gelatinase granules, respectively [47]. Like all proteases, tight regulation is required to ensure localisation of the enzymes to the area of infection; otherwise tissue damage can occur. We observed that, even under the same stimulation conditions, NE and MMP-9 release were differentially regulated, that is, a deficient fMLF induced MMP-9 response but an augmented fMLF induced NE response in neutrophils from well-controlled asthmatics. This differential regulation could be due to the location of these products in different types of granules and their differing propensity to be released from neutrophils under certain stimulation conditions. However, further investigation of these mechanisms was outside the scope of this study and could be the subject for future studies.

Interestingly, we found similar neutrophil responses between patients with suboptimally controlled asthma and well-controlled asthma, particularly with deficient fMLF induced CXCL8 and MMP-9 release. Neutrophil function is known to decline with age and these changes include decreased phagocytic ability [48, 49], reduction in degranulation [50], and reduced capacity to generate reactive oxygen species [48]. In previous work we found that neutrophils from asthmatics with a mean age of 35 years had a greater propensity to release CXCL8 with R848 stimulation compared to nonasthmatic controls; however, other stimulants which included fMLF were similar between the two groups [25]. In this study where participants had a mean age of 62 years, we did not observe enhanced R848 induced CXCL8 from neutrophils, rather similar levels between both asthmatic groups and healthy controls. In addition, we noted deficient CXCL8 and MMP-9 release with fMLF stimulation which may suggest that decline in neutrophil function is greater in those with disease, particularly in recognition of bacteria.

Studies have reported CXCL8 levels in the airways inversely correlate with FEV_1_ in asthmatic individuals [3, 51, 52]. These studies measured CXCL8 in the bronchial alveolar lavage (BAL) and induced sputum which indicate the total inflammatory mediator load in the airways but give little indication of the source. In this study we found that basal CXCL8 release from neutrophils does not correlate with lung function, suggesting that neutrophils may not be the main source of this cytokine in BAL. However, fMLF induced CXCL8 positively correlated with both FEV_1_ and FEV_1_/FVC. fMLF stimulates neutrophils via the FPR1 receptor. Interestingly annexin A1 also activates the fMLF receptor [53]. As corticosteroid induced annexin A1 is a major anti-inflammatory mechanism it is interesting to speculate that the responsiveness to fMLF may also indicate steroid insensitivity.

Our study has several limitations; the number of participants is small and the patients with suboptimal asthma control may have been quite heterogenous. Few participants had an ACQ > 1.5, which is the cut point used to be confident that asthma control is poor [30]. As we do not have eosinophil counts there is potential that the suboptimally controlled asthma group could contain steroid refractory eosinophilic asthmatics. These patients are distinct from neutrophilic refractory asthmatics as they have eosinophilia despite high dose steroid therapy and respond to anti-IL-5 antibody therapy [54, 55]. Detailed phenotyping and endotyping of asthma are areas of interest to help personalise treatment options [56]. Our study is limited in this respect and future studies with larger population groups could address this question. It is also becoming clear that neutrophils are a nonhomogenous population of cells that carry out various functions ranging from the classical proinflammatory response to an immune modulatory response [57]. Investigation in this area was out of the scope of our study but would be of interest to closely investigate neutrophil subtypes in different asthmatic populations.

In conclusion, no significant differences were seen in neutrophil function between patients with well- and suboptimally controlled asthma and therefore it is unlikely that neutrophil dysregulation drives asthma control. However, neutrophils from people with asthma appear to have different responses to pathogenic stimuli compared to healthy controls. This dysfunction may contribute to persistent or greater susceptibility to infection in asthmatics and is likely to be associated with airway obstruction.

## Figures and Tables

**Table 1:** Patient characteristics of healthy controls and asthmatics.

	Healthy controls	Asthma, well-controlled	Asthma, suboptimally controlled
*N*	9	11	7
Age years, mean (±SEM)	60.22 (±5.12)	62.64 (±3.97)	62.57 (±4.02)
Gender (M/F)	6/3	7/4	5/2
ACQ, mean (±SEM)	0 (±0)	0.32 (±0.08)^*∗*^	1.33 (±0.17)^*∗∗∗∗*/####^
FEV_1_% pred. (±SEM)	92.78 (±5.98)	80.55 (±7.71)	70.29 (±8.09)
FEV_1_/FVC% pred. (±SEM)	101.8 (±1.65)	88.36 (±4.51)^*∗*^	77.43 (±5.27)^*∗∗*^
Short-acting *β*_2_ agonist use (Y/N)	0/9	9/0	7/0

^*∗*^ *p* < 0.05, ^*∗∗*^ *p* < 0.01, and ^*∗∗∗∗*^ *p* < 0.0001 compared to healthy controls.

^####^ *p* < 0.0001 compared to patients with asthma with good symptom control.

ACQ: asthma control questionnaire.

FEV_1_: forced expiratory volume in 1 second.

FVC: forced vital capacity.

**(a):** 

Healthy controls			
Age	Sex	Smoking status	Pack years
66	Male	Nonsmoker	0
66	Male	Nonsmoker	0
62	Female	Ex-smoker	15
70	Male	Nonsmoker	0
63	Female	Nonsmoker	0
67	Male	Nonsmoker	0
61	Female	Nonsmoker	0
67	Male	Nonsmoker	0
20	Male	Nonsmoker	0

**(b):** 

Asthma, well-controlled^*∗*^					
Age	Sex	Smoking status	Pack years	Daily ICS BDP-HFA equivalent (*μ*g/day)	ICS in last 12 months in BDP-HFA equivalent (*μ*g/day)
69	Female	Nonsmoker	0	0	200 (during exacerbations)
71	Male	Ex-smoker	18	200	—
75	Male	Nonsmoker	0	0	—
63	Male	Ex-smoker	0.5	1000	2000 (when needed)
52	Male	Nonsmoker	0	0	200^†^ (exercise only)
61	Female	Nonsmoker	0	0	200 (in the last 12 months, ceased 3 months ago)
63	Male	Current smoker	43	0	200 (in high humidity, not used in the past months)
73	Male	Ex-smoker	21	1000	—
28	Male	Nonsmoker	0	125	—
69	Female	Nonsmoker	0	100^†^	200^†^ (when sick)
65	Female	Nonsmoker	0	250	500 (when sick)

**(c):** 

Asthma, suboptimally controlled^*∗*^					
Age	Sex	Smoking status	Pack years	Daily ICS BDP-HFA equivalent (*μ*g/day)	Oral steroid use in last 12 months
62	Male	Ex-smoker	0.2	800	Yes
71	Female	Ex-smoker	0.6	400	Yes
74	Male	Ex-smoker	4.5	1000	No
47	Male	Nonsmoker	0	1000	No
73	Female	Nonsmoker	0	1000	Yes
52	Male	Ex-smoker	7.15	200	No
59	Male	Nonsmoker	0	500	No

^*∗*^See Section 2 for inclusion criteria.

^†^ICS-only inhaler; all other patients using ICS/LABA consistent with Australian prescribing trends [37].

BDP-HFA: beclometasone dipropionate (hydrofluoroalkane propellant).

ICS: inhaled corticosteroid.

## Abbreviations

ACQ: Asthma control questionnaire
BDP-HFA: Beclometasone dipropionate (hydrofluoroalkane propellant)
CXCL8: Interleukin 8
FEV_1_: Forced expiratory volume in 1 second
fMLF: f-Met-Leu-Phe
FVC: Forced vital capacity
ICS: Inhaled corticosteroid
LPS: Lipopolysaccharide
MMP-9: Matrix metalloproteinase-9
NE: Neutrophil elastase
RV: Rhinovirus.
